# A systematic review of the cardiotoxicity of methadone

**DOI:** 10.17179/excli2015-553

**Published:** 2015-05-05

**Authors:** Samira Alinejad, Toba Kazemi, Nasim Zamani, Robert S. Hoffman, Omid Mehrpour

**Affiliations:** 1 Atherosclerosis and Coronary Artery Research Center, Birjand University of Medical Sciences,Birjand, Iran; 2 Department of Clinical Toxicology, Loghman Hakim Hospital, Shahid Beheshti University of Medical Sciences, Tehran, Iran; 3 Division of Medical Toxicology, Ronald O. Pereleman Department of Emergency Medicine, New York University School of Medicine, New York, NY, USA; 4 Medical Toxicology and Drug Abuse Research Center (MTDRC), Birjand University of Medical Sciences (BUMS), Pasdaran Avenue, Birjand, 9713643138 Iran

**Keywords:** methadone, toxicity, heart, ECG, torsade de pointes, QT interval

## Abstract

Methadone is one of the most popular synthetic opioids in the world with some favorable properties making it useful both in the treatment of moderate to severe pain and for opioid addiction. Increased use of methadone has resulted in an increased prevalence of its toxicity, one aspect of which is cardiotoxicity. In this paper, we review the effects of methadone on the heart as well as cardiac concerns in some special situations such as pregnancy and childhood. Methods: We searched for the terms methadone, toxicity, poisoning, cardiotoxicity, heart, dysrhythmia, arrhythmia, QT interval prolongation, torsade de pointes, and Electrocardiogram (ECG) in bibliographical databases including TUMS digital library, PubMed, Scopus, and Google Scholar. This review includes relevant articles published between 2000 and 2013. The main cardiac effects of methadone include prolongation of QT interval and torsade de pointes. Other effects include changes in QT dispersion, pathological U waves, Taku-Tsubo syndrome (stress cardiomyopathy), Brugada-like syndrome, and coronary artery diseases. The aim of this paper is to inform physicians and health care staff about these adverse effects. Effectiveness of methadone in the treatment of pain and addiction should be weighed against these adverse effects and physicians should consider the ways to lessen such undesirable effects. This article presents some recommendations to prevent heart toxicity in methadone users.

## Introduction

Acute overdose is one of the main complications of abuse and causes of mortality in opioid addicted patients (Afshari et al., 2007[2]; Ayatollahi et al., 2011[12]). Approximately, eight million people are substance abusers worldwide, most of whom, are from southeast and southwest Asia (Justo et al., 2006[67]). Iran has the highest rate of opioid addiction in the world (Karrari et al., 2012[70]; Mehrpour, 2012[101]; Mehrpour and Sezavar, 2012[103]). This country appears to be involved in both traditional and modern drug use and abuse problems and has an important role in the transportation of opium from Afghanistan to western countries (Day et al., 2006[31]; Karrari et al., 2013[69]). While in western countries, alcohol, cannabis, methamphetamine, and heroin are the most common abused drugs, in Iran opium remains the most commonly abused drug with opium poisoning and overdose being the major cause of drug-related hospital admissions (Movaghar et al., 2005[105]; Ayatollahi et al., 2011[12]; Jafari et al., 2010[63]; Koushesh and Afshari, 2009[78]; Taghaddosinejad et al., 2011[152]).

Methadone, a synthetic opioid, was first produced in 1937 in Germany during world war II. The US Food and Drug Administration (FDA) approved it as an analgesic in 1947 (Ehret et al., 2007[36]; Noorzurani et al., 2009[109]; Shields et al., 2007[141]; Stimmel, 2011[150]). Methadone is an agonist of mu receptors (Izadi-Mood et al., 2008[62]) that has been used as an alternative treatment in the control of opioid dependency since the 1960s (Justo et al., 2006[67]). Methadone Maintenance Treatment (MMT) is the common for methadone treatment in opioid addiction (Izadi-Mood et al., 2008[62]) and is the best choice for treatment of opioid dependence (Pani et al., 2011[111]; Peles et al., 2007[118]). The use of MMT began in the US in 1964 when Dole and Nyswander first used this synthetic opioid for narcotic addiction (Palmiere et al., 2011[110]) and is an appropriate option for patients with a history of long-term opioid addiction when abstinence and other therapies have failed (Kobek et al., 2009[76]).

At least 750000 patients are on MMT worldwide (Ehret et al., 2007[36]). In America and Australia, about 250000 and 23300 patients are on MMT, respectively (Thanavaro and Thanavaro, 2011[154]; Zador and Sunjic, 2000[167]). Also, methadone is the most common prescribed drug for opioid dependence in Ireland (Roy et al., 2012[130]; Teichtahl et al., 2004[153]). In Europe, use of MMT ranges from 6-22 % in the United Kingdom (UK) to 41-86 % in Spain (Justo et al., 2006[67]). The higher incidence of methadone poisoning in Iran may be due to MMT, which has only been started in recent years and has increased access to methadone (Ayatollahi et al., 2011[12]).

Methadone has different applications in the clinics. It is highly lipid-soluble and can therefore be administered once daily; it has long elimination half-life and is metabolized by liver (renal function does not interfere with half-life), and is an antagonist of the N-methyl-D-aspartate (NMDA) receptor causing reducing neuropathic pain (Ehret et al., 2007[36]). Methadone is different from other drugs in that it causes less stupor and does not interfere with mental and physical activities causing the patients to have better social relationships (Kobek et al., 2009[76]). MMT has five main benefits: reduction in the use of illegal substances, decreasing viral transmission through reduced injection of drugs, declining deaths due to excessive use of drugs, improvement in physical and mental health, and diminishing criminal activities (Fahey et al., 2003[40]). Other important uses of methadone are obviating moderate to severe pain (Palmiere et al., 2011[110]) and resistant cough in patients with lung cancer (Izadi-Mood et al., 2008[62]).

Although methadone is a safe drug, overdoses have been reported (Cruciani 2008[28]; Paulozzi et al., 2009[116]). It also causes some side effects on central nervous system, skin, gastrointestinal tract, and urogenital and cardiovascular systems (Izadi-Mood et al., 2008[62]). One of the adverse events of methadone is its cardiotoxicity (Kumar, 2010[84]). Indeed, increased QT dispersion, QT interval prolongation, and torsade de pointes (TDP) -a life-threatening arrhythmia- are all reported in patients treated with methadone (Cruciani 2008[28]; Price et al., 2013[122]). These cardiovascular side effects were so prominent when a derrivative Levacetyl methadol (LAAM) or Orlaam (Deamer et al., 2001[33]) which caused LAAM was introduced, that it had to be eliminated from European markets (Deamer et al., 2001[33]; Guay, 2009[52]; Kumar, 2010[84]).

The goal of this paper is to review, in detail, the cardiotoxic potential of methadone, its mechanisms and possible treatments, and provide recommendations for physicians and healthcare staff to prevent it.

## Materials and Methods

We searched for the terms methadone, toxicity, poisoning, cardiotoxicity, heart, dysrhythmia, arrhythmia, QT interval prolongation, torsade de pointes, and electrocardiogram (ECG) in bibliographical databases including TUMS digital library, PubMed, Scopus, and Google Scholar. This review includes relevant articles published between 2000 and 2013. We chose only the articles in this time frame because we wanted to work as much as possible on newer articles and also we did not find any different related papers before this time. We included not only human studies but also animal works in our study. 

### Pharmacology of methadone

Methadone is the drug of choice for the treatment of opioid addiction due to its special pharmacokinetics (Strain, 2002[151]). It is also a good alternative to morphine and other opiate analgesics in the treatment of severe chronic pain (Kumar, 2010[84]). The average bio-availability of methadone (about 80 %) is higher than other opioids and its elimination half-life is long ranging from 7 to 65 hours (Fredheim et al., 2008[45]). Because of high solubility, 98 % of methadone that has reached the central compartment is quickly transported to tissues, especially to the liver, kidneys, lungs, and in small proportion, to the brain. Almost 1-2 % remains in the blood with 60-90 % bound to plasma proteins (mostly alpha_1_-acid glycoprotein) (Corkery et al., 2004[26]; Ferrari et al., 2004[43]; Gallagher 2009[48]; Shir et al., 2001[142]). Methadone passes the placenta and is also secreted in the breast milk.

Methadone hydrocholoride is a racemic combination of two enantiomers (R and S), which differ in diffusion, elimination, and effects. The R-enantiomer has 10-times the potency at the opioid receptor in vitro, a longer elimination half-life, and a higher total volume of distribution. It is responsible for almost all analgesic effects of the drug (Ehret et al., 2007[36]).

Certain cytochrome P450 enzymes in liver or other organs metabolize methadone. Methadone is mainly metabolized by Cytochrome P450 3A4 (CYP3A4) and (CYP2D6) with the latter having a secondary role (Pimentel and Mayo, 2008[121]; Sticherling et al., 2005[149]). Expression of CYP3A4 is the main factor causing diversity of methadone bioavailability in different people. It also participates in the metabolism of other drugs including benzodiazepines, calcium antagonists, macrolides, rifampin, and anticonvulsants. On the other hand, some drugs including ketoconazole, fluoxetine, and grapefruit juice in the large amount inhibit CYP3A4 activity and may cause the risk of drug-drug interaction. In Iran, the frequency of CYP2D6 metabolizers is up to 12 % of the population; thus, we expect people in this region to be more susceptible to opioid effects such as dependency and sedation (Mehrpour, 2013[100]).

The kidneys are the main organs in the excretion of methadone and its metabolites (15-40 % during the first 24 hours). Fecal excretion is responsible for 20-40 % of the drug elimination (Ferrari et al., 2004[43]). In a retrospective review conducted on 185 patients with cancer pain to measure the clearance of methadone, it was shown that a mean methadone clearance was 186 mL/min and a mean elimination half-life was 61.8 hours (Karir, 2002[68]).

Methadone is preferably orally administered (Palmiere et al., 2011[110]) and is available as an oral solution (1-2 mg/mL), tablets (5-10 mg), dispersible tablets (40 mg), and injectable solutions (10 mg/mL). Methadone, has also a local use in the form of mouth wash for the treatment of painful oral ulcers and in powder as an analgesic on open wounds (Palmiere et al., 2011[110]).

In order to reduce the debate of suitable methadone dosing, low-dose, intermediate-dose, and high dose of methadone have been defined as doses < 50 mg/day, 50-100 mg/ day, and > 100 mg/day, respectively (Strain, 2002[151]). The recommended dose for the relief of severe pain is 2.5-10 mg every 3-4 hours. This dose is 60-80 mg/day for methadone maintenance varying from 30 to 120 mg/ day (Couper et al., 2005[27]). The treatment, toxic, and fatal serum concentrations of methadone range between 0.075 and 1.1 microgram/mL, 0.2 and 2 microgram/mL, and 0.4 and 2.8 microgram/mL, respectively (Kobek et al., 2009[76]) and vary dramatically with dependence.

### Cardiotoxicity

Experimental studies have found that methadone can affect many cardiac function parameters through various mechanisms (Sánchez Hernández et al., 2005[133]). Despite being considered to be safe, there have been some cases of cardiotoxicity leading to sudden death (Chugh et al., 2008[23]; Cruciani, 2008[28]).

### QT prolongation

The QT interval represents the required time for ventricular depolarization and repolarization (Spevak et al., 2012[146]) measured from the beginning of QRS complex to the end of the T wave. This interval changes with heart rate and is often corrected for this yielding a corrected QT (QTc) (Atkinson et al., 2007[11]; Wilcock and Beattie, 2009[164]). A QTc less than 450 and 430 msec is normal in females and males, respectively (Goldenberg et al., 2006[51]; Kornick et al., 2003[77]). Drugs such as methadone and cocaine can cause QT prolongation through the direct effects on the resting membrane potential (Wallner et al., 2008[161]). Methadone and QT prolongation is a subject of international debate (Krook et al., 2004[83]; Mayet et al., 2011[99]). This relation was elucidated almost four decades ago (Mayet et al., 2011[99]). It has been shown that daily use of methadone can increase QT interval by 12 msec (Abramson et al., 2008[1]; Krantz et al., 2005[81]).

As mentioned previously, early studies reported a relationship between methadone use and QT prolongation. Several cross-sectional studies have since confirmed this association (Anchersen et al., 2009[4]); Ehret et al. (2006[37]), compared drug-using patients receiving methadone with those who did not receive it among all patients hospitalized over a 5-year period in a tertiary care hospital. Of 167 methadone patients, QT prolongation was detected in 16.2 %. They concluded that QT prolongation was common among methadone maintenance patients. Wedam et al., 2007[162], compared QT interval effects of methadone, levomethadyl, and buprenorphine in a randomized trial. Baseline QTc was similar in the three groups. The levomethadyl and methadone groups were significantly more likely to show QTc greater than 470 to 490 msec or an increase from baseline in QTc of greater than 60 msec.

### QT interval dispersion

Methadone can increase QT dispersion in addition to QT interval (Hassanian-Moghaddam et al., 2014[55]). QT dispersion reflects the variety of QT intervals in the 12-lead ECG and is indicative of abnormal cardiac repolarization (Krantz et al., 2005[81]; Somberg and Molnar, 2002[145]). The normal range for QT dispersion is 30-60 msec (Krantz et al., 2005[81]).

Krantz et al., investigated the effects of methadone on QT interval dispersion in 118 patients who had joined the MMT facility. Twelve-lead ECGs were performed at both baseline and 6 months after the start of methadone therapy. Mean baseline QT dispersion was 32.9 msec, which increased to 42.4 msec after 6 months of therapy. The QTc increased by a similar magnitude. No QT dispersion value exceeded 100 msec. They concluded that methadone could increase QT dispersion as well as QT interval (Krantz et al., 2005[81]).

### Torsade de pointes (Tdp)

QT prolongation is common in patients on MMT; however, in patients with profoundly prolonged QT (≥ 500 msec), undesirable complications may occur that expose the patients to development of Tdp (Almehmi et al., 2003[3]; Drew et al., 2010[34]; Fredheim et al., 2006[46]; Gallagher et al., 2008[48]; Priori et al., 2003[123]; Thanavaro and Thanavaro, 2011[154]; Walley et al., 2013[160]). Tdp is an abnormal heart rhythm that causes regular and wide polymorphic QRS complex tachycardia twisting around the iso-electric baseline (Tdp is a French word meaning "twisting of the points”). This rhythm is potentially fatal because it may progress to ventricular fibrillation and therefore should not be left untreated (Anchersen et al., 2010[5]; Tünsmeyer et al., 2012[155]). The most common cause of Tdp is drug use (Roden, 2008[128]). Dessertenne first described Tdp in 1966 (Chiang, 2006[22]; Ehret et al., 2007[36]) and Krantz et al. (2002[80]), reported the association between methadone and Tdp for the first time in 2002. The increase in the dosages of the drug in recent years has resulted in more cases of this abnormal rhythm. Another possibility is that patients taking methadone use newer drugs and therefore are at risk of dangerous drug interactions including prolongation of the QT interval when they use them in combination with their methadone.

Methadone inhibits the Human Ether-a-go-go Related Gene (hERG) and causes QTc prolongation and development of Tdp (Eap et al., 2007[35]; Esfahani et al., 2012[38]; Sekine et al., 2007[140]; Zünkler and Wos-Maganga, 2010[169]). hERG, first identified in 1994, is located on chromosome 7 and codes for the potassium ion channel which intercedes repolarization of the cardiac action potential (Parikh et al., 2011[112]). This gene is expressed in multiple tissues and cells including neural, smooth muscle, and tumor cells. However, it is most highly expressed in the heart (Sanguinetti and Tristani-Firouzi, 2006[135]; Smith et al., 2002[144]).

The action potential of human ventricular myocytes can be divided into five distinct phases. Inward sodium current triggers a rapid membrane depolarization (phase 0). Repolarization occurs in three phases; phase 1 which proceeds rapidly and lasts a few milliseconds; phase 2 called the plateau which is a prolonged because the K^+ ^currents activated during this phase are slow to activate and have a reduced conductance at positive trans-membrane potentials; phase 3 in which the action potential terminates, and phase 4 in which the membrane returns to its resting level. The most important component of phase 3 is the rapid delayed rectifier K^+^ current (*I*Kr) conducted by hERG channels (Sanguinetti and Tristani-Firouzi, 2006[135]).

hERG is highly susceptible to pharmacological blockade (Sanguinetti and Mitcheson 2005[134]). Methadone blocks the hERG channels at concentrations close to those clinically achieved (Chugh et al., 2008[23]). This blockage in turn prolongs the end part of cardiac action potential and finally reprieves repolarization represented as QTc interval prolongation (Esfahani et al., 2012[38]; Fonseca et al., 2009[44]; Hassnain-Moghaddam et al., 2013[55]). Methadone's R-isomer inhibits the hERG channel less than the S-isomer (Lin et al., 2008[92]). Methadone can cause QT prolongation and Tdp with other mechanisms, as well, including producing negative chronotropic effects via Ca^++ ^channel antagonism and anti-cholinesterase effect. The bradycardia induced by these mechanisms makes the patient more susceptible to Tdp (Esfahani et al., 2012[38]). Accepted risk factors for QT prolongation and Tdp are low K^+^, Mg^++^, or Ca^++^ concentrations, diabetes mellitus, thyroid or pituitary insufficiency, cardiomyopathy, recent myocardial infarction (MI), sinus bradycardia, certain drugs, toxins (organophosphates, insecticides, and heavy metals), female gender, older age, subarachnoid hemorrhage, starvation, genetic susceptibility, obesity, alcoholism, and cirrhosis (Bednar et al., 2001[14]; Gupta et al., 2007[53]; John et al., 2010[65]; Kobek et al., 2009[76]; Laqueille et al., 2012[88]; Pasquier et al., 2012[113]; Raschi et al., 2009[126]; Schmidt, 2005[137]; Vieweg et al., 2013[156]; Viskin et al., 2003[158]).

Patients on MMT often receive concomitant medications for psychiatric disorders and infections such as Human Immunodeficiency Virus (HIV) which can raise the risk of drug-drug interactions (Lüthi et al., 2007[93]). In methadone users, drug-drug interaction results in TdP via a pharmacodynamic combined effect and causing an alteration of metabolism. Some medications can cause QTc prolongation per se (including amiodarone, chloroquine, clarithromycin, erythromycin, haloperidol, lithium, sotalol, terfenadine, and venlafaxine as well as grapefruit juice). They, when given with methadone, may increase the risk of development of TdP. Among them are some commonly used medications such as amiodarone, chlorpromazine, cisapride, clarithromycin, droperidol, erythromycin, haloperidol, pentamidine, pimozide, procainamide, quinidine, sotalol, thioridazine, quinolones, and antifungals. With regard to the pharmacokinetics, methadone is mainly metabolized by CYP3A4 enzyme and to a lesser extent by CYP2B6 and CYP 2D6. Induction or inhibition of these enzymes by any means including certain drugs can increase or decrease the serum concentrations of methadone. HIV antivirals (e.g., indinavir, nelfinavir, and ritonavir), antibiotics and antifungals such as clarithromycin, itraconazole, and ketoconazole strongly inhibit CYP3A4 while efavirenz induces it (Prosser et al., 2008[124]).

Nair et al. (2008[106]) introduced a 56-year-old man receiving methadone brought to their emergency department because Tdp developed after using ciprofloxacin in whom CYP1A and CYP3A enzymes likely caused a pharmacokinetic reaction. Sánchez Hernández et al. (2005[133]) reported four cases of Tdp during methadone treatment all of whom were on MMT. Three of them were HIV-infected and one received antiretroviral therapy. Two patients had low serum potassium concentrations. In fact, these patients had some associated risk factors that caused QT interval prolongation. Justo and colleagues (2006[67]) reviewed all the publications on methadone-associated Tdp in addicted patients to identify the situations leading to this complication. All patients had at least one risk factor for Tdp including high-dose methadone, concomitant use of agents that increased serum methadone concentrations, HIV infection, hypokalemia, female gender, liver cirrhosis, or renal failure. Kuryshev and associates (2010[85]) evaluated the cardiac risk in co-administration of methadone and diazepam. They found that diazepam alone did not prolong the QT interval; but, its concomitant use with methadone interacted with Na^+^ channels and caused hERG K^+^ channel block. Winton and Twilla (2013[165]) reported a 47-year-old addicted man who was on methadone and azithromycin for an upper respiratory tract infection and experienced a fatal arrhythmia. Schmittner et al. (2009[138]) evaluated the ECG effects of co-administration of lofexidine and methadone in 14 participants dependant to opioids. There were significant changes in four ECG parameters; decreased heart rate and increased PR, QRS complex, and QT intervals. In three participants, QTc prolongation was clinically significant (it increased by > 40 msec from baseline).

Reinhold et al. (2009[127]) reported a 57-year-old man with comorbid conditions who developed Tdp after use of methadone and voriconazole. The probable cause was voriconazole-inhibited methadone metabolism via cytochrome P450 isoenzymes (CYP2B6). Krantz and colleagues (2005[82]) introduced a patient on MMT who developed syncope due to Tdp hours after cocaine use. They found that cocaine and methadone prolonged QT interval via the same mechanism.

### Methadone dose dependency in QT prolongation and Tdp

Ideal methadone dosage in treatment of opioid dependence is a matter of debate (Liao et al., 2013[91]). Several large studies have been performed to evaluate the relationship between the dose of methadone and QTc prolongation. Some have reported a direct relationship (Table 1(Tab. 1); References in Table 1: Krantz et al. (2003[79]); Routhier et al. (2007[129]); Walker et al. (2003[159]); Ehret et al. (2006[37]); Cruciani et al. (2005[29]); Martel et al. (2005[98]); Thanavaro and Thanavaro (2011[154])), while others could not find such an association (Table 2(Tab. 2)Krantz et al., 2005[81]; References in Table 2: Maremmani et al. (2004[96]); Peles et al. (2007[118]); Huh and Park, 2010[57]; Pearson and Woosley, 2005[117]; Roy et al., 2012[130]). There are several case reports and data on methadone-induced QT prolongation in a dose-dependent manner that have caused considerable concern for practitioners in addiction medicine (Butler et al., 2011[17]). Oral recommended doses range from 60 to 100 mg per day. It has been shown that use of a mean dose of 100 mg or more of methadone leads to significant dose-dependent QTc prolongation in these patients (Chang et al., 2012[21]). Krantz et al. (2003[79]), investigated the relationship between the daily dose of methadone and QTc interval in methadone-treated patients who developed Tdp. They concluded that daily methadone dose correlated positively with the QTc interval. Routhier and associates (2007[129]) presented a 52-year-old woman without any underlying cardiac disease who developed QTc prolongation and Tdp secondary to high-dose methadone therapy. This case report suggested that methadone induced QTc prolongation and Tdp in a dose-dependent manner. Walker and associates (2003[159]) published a report of three cases of Tdp in patients on daily doses of methadone exceeding 600 mg, two of whom, presented with syncope and one with respiratory distress. Each of the three was on other medications that inhibited the metabolism of methadone. Extra caution should be given for arrhythmias when high dosages of methadone (> 600 mg/day) are used, especially in patients on other drugs that can interact with the CYP3A4 isoenzyme system. Ehret et al. (2006[37]) investigated the frequency of QT prolongation in MMT patients hospitalized in a tertiary center and identified associated risk factors. Methadone dose, CYP 3A4 inhibitors, potassium concentration, and liver function contributed to QT prolongation and QTc length was weakly but significantly associated with methadone daily dose. In the study by Cruciani and colleagues (2005[29]), the median methadone dose was 110 mg/day. Significant dose-response was observed in males on methadone for less than 12 months. Martell and assistants (2005[98]) carried out a study on 160 patients, all of whom were initiated on 30-mg oral methadone with subsequent 10-mg incremental increases. They observed that there was a positive correlation between serum methadone concentration and the prolongation of QTc interval. Thanavaro and Thanavaro (2011[154]), reported a case of methadone-induced Tdp who was on high dose of the drug (110 mg/day) without using any concomitant medications. They concluded that methadone dose should be adjusted or the drug should be switched to an alternative one and ECG should be repeated in case of QTc prolongation. Although several studies indicated an association between methadone dose and QTc interval, other investigations did not detect such effect (Krantz et al., 2005[81]). QT interval prolongation has been detected with daily doses below 65 mg, as well (Huh and Park, 2010[57]).

Maremmani et al. (2004[96]) evaluated abnormal QTc interval in 83 heroin addicts who were on long-term MMT and received methadone dosages between 10-600 mg/day. Almost 83 % of them had QT prolongation more than the reference values. No correlation existed between QT prolongation and methadone dosages. Peles and associates (2007[118]) studied 138 patients on MMT with methadone dose of 40-290 mg/day. An ECG was done and a serum methadone concentration was determined about 24 hours after the last oral methadone dose. It was shown that methadone dose and serum concentrations did not correlate with QTc. 

Huh and Park (2010[57]) conducted a study on 130 patients with 90 patients in the methadone and 40 patients in the control group. Heart rate, QT interval, and QTc were recorded. The patients’ demographics, methadone dose and serum concentration, duration of methadone use, and past medical history were collected. QTc interval was significantly longer in the methadone group. QTc interval was not associated with methadone dose (P = 0.9) and serum concentration or duration of treatment. Pearson and Woosley (2005[117]) reviewed and analyzed adverse events (QT prolongation and Tdp reported to FDA from 1969 to 2002) to determine the patients’ characteristics, dosages of methadone, and outcomes of methadone-treated patients. Analysis of the cases showed that QT prolongation and Tdp could occur over a wide range of dosages even those recommended for treatment of addiction. Roy and colleagues (2012[130]) evaluated the correlation between QT interval and methadone dose. Mean methadone dose was 80.4 ± 27.5 mg in their study. They concluded that prolongation of QT interval was seen even in patients receiving low doses of methadone without any dose-response relationship.

No cut-off level for safe dose has been identified. Although some studies have detected positive association between methadone dose and QTc prolongation, the relationship is usually stronger with additional factors such as gender, duration of treatment, and concomitant drug use (Huh and Park, 2010[57]).

### U-waves

Methadone can cause pathological U waves (larger than the T wave) and impending Tdp (John et al., 2010[65]). Drug-induced or prolonged QT syndromes can create U waves. In a study on the effects of methadone and buprenorphine on ECG, methadone subjects were significantly more likely to have U waves (Athanasos et al., 2008[10]).

### Ventricular bigeminy

Ventricular bigeminy may be due to different mechanisms involving a disturbance of impulse form/conduction or both (Langendorf et al., 1955[87]). Scholler et al. (2011[139]) demonstrated ventricular bigeminy in a Caucasian woman with concomitant administration of methadone, voriconazole, and esomeprazole. She had high plasma concentrations of voriconazole and methadone. It was concluded that a pharmacokinetic interaction between methadone and voriconazole was reinforced by the addition of esomeprazole.

### Tako-Tsubo Syndrome

Stress cardiomyopathy (Tako-Tsubo syndrome) is defined as left ventricular functional and ECG changes that mimic acute myocardial infarction without any evidence of involvement of coronary arteries. It is more common in postmenopausal women and an acute medical illness or emotional/physical stress may typically trigger this syndrome (Lemesle et al., 2010[90]; Saiful et al., 2010[132]). It has been demonstrated that huge release of catecholamines (maybe due to opioid withdrawal) may be responsible for development of this cardiomyopathy. Only two cases of Taku-Tsubo syndrome have been reported in relation with acute opioid withdrawal. Lemesle et al. (2010[90]), reported the first case as a result of methadone withdrawal secondary to drug-drug interaction.

### Brugada-like syndrome

Brugada-like syndrome is another condition in methadone users predisposing the patients to life-threatening ventricular tachycardia and sudden cardiac death. The disease is due to a mutation in the cardiac sodium channel SCNSA gene in one-fourth of the cases. Although the patients may have normal ECGs, coved-type ST elevation in precordial leads V1–V3 is characteristic of the disease. Agents causing Brugada-like ECG include fever, cocaine and methadone, sodium channel blockers (e.g. propafenone, procainamide, flecainide, bupivacaine, lidocaine, and tricyclic antidepressants), propofol, and electrolyte imbalances (Deamer et al., 2001[33]). Srivatsa and colleagues (2005[147]) reported a case of consecutive appearance of Brugada and long QT patterns on ECG in a patient receiving methadone. Junttila et al conducted a multi-center observational trial on 47 patients presenting with typical Brugada-like ECGs during an acute medical event, 16, 26, and 5 cases were due to febrile episodes, drugs, and electrolyte imbalances, respectively. Fifty-one percent had severe arrhythmias with 38 % leading to sudden cardiac death and 6 % developing ventricular tachycardia and syncope. One of the victims of sudden cardiac death was receiving methadone (Junttila et al., 2008[66]).

### Methadone and coronary artery disease

Cardiovascular diseases are the most common causes of death in both genders (Kazemi, 2012[73]; Kazemi et al., 2011[74]) and have been noticed to lead to morbidity and mortality in MMT clinics. Although long-term methadone exposure may relieve the fatal outcomes of coronary diseases (Marmor et al., 2004[97]; Patane et al., 2007[114]; Safaei, 2008[131]), opiate-related ischemia has been described in some cases. Backmund and associates presented a 22-year-old addicted man who suffered myocardial infarction after concomitant use of methadone and dihydrocodeine (Backmund et al., 2001[13]). Patane and assistants (2007[114]) presented a case of acute myocardial infarction in a chronic methadone user after aspirin use (due to the paradoxical activation of major platelet receptors after administration of aspirin).

### Methadone and hemodynamic effects

The cardiovascular toxicity of opioids causes significant morbidity and mortality in overdose; but, the hemodynamic effects of opioids reported in animal and human studies are contradictory (Afshari et al., 2007[2]). Afshari et al. (2007[2]) performed a prospective observational study of patients admitted to hospital following an overdose of methadone, dihydrocodeine, or low-dose paracetamol (10 each). They observed that dihydrocodeine and methadone significantly reduced peripheral and aortic systolic, mean and end systolic pressures. Both significantly decreased peripheral pulse pressure, but only methadone decreased aortic blood pressure. Dihydrocodeine reduced systemic and aortic diastolic blood pressure, an effect not induced by methadone. Methadone reduced peripheral pulse pressure. Augmentation index and heart rate, however, did not change. Both opioids decreased arterial oxygen saturation. It was suggested that dihydrocodeine and methadone had a significant effect on central and peripheral hemodynamic when overdosed. Anderson and Alvarado (2003[6]) assessed acute hemodynamic effects of intravenous methadone in 21 patients all of whom were scheduled for cardiac surgery. They found that there were no significant changes in any hemodynamic parameter at any time point. This study indicates that no adverse hemodynamic effects accompany a 20-mg intravenous bolus of methadone.

### Use of methadone in pregnancy

Opioid dependence is a major public health problem during pregnancy. A recent study showed that 2.6 % of pregnant women in the United States had positive opioid tests at time of admission for delivery. It is estimated that in Europe 30000 opioid-addicted women become pregnant annually (Schmid et al., 2010[136]).

Methadone is the routine substitution therapy for heroin-addicted pregnant women (Navaneethakrishnan et al., 2006[107]; Burns et al., 2010[16]). It easily crosses the placenta and the final concentration of methadone in the umbilical cord is one-fourth of that in maternal serum (Schmid et al., 2010[136]). Pregnant women on MMT have better prenatal care in comparison with the untreated women (Ramirez-Cacho et al., 2006[125]), which confers higher birth weights and less obstetric complications, preterm births, and neonatal morbidity. Despite these benefits, common harmful perinatal complications have also been reported in methadone-exposed pregnant women (Cleary et al., 2012[25]). Methadone use by mother changes fetal heart rate (FHR) patterns; for example, reduces the baseline heart rate and variability and increases the possibility of a non-reactive non-stress test (Jansson et al., 2005[64]; Leeman et al., 2011[89]). Ramirez-Cacho (2006[125]) and colleagues compared intrapartum FHR tracings from 56 mothers in their 36^th^ gestational age and on MMT with a healthy control group matched for maternal age, parity, gestational age, and ethnicity. It was concluded that chronic maternal methadone treatment affected intrapartum FHR patterns by reducing the variability, baseline, and proportion of accelerations during the first stage. Schmid and colleagues (2010[136]) evaluated the FHR by Doppler ultrasound between 11+0 and 13+6 gestational weeks in methadone-using mothers and a healthy control group. They concluded that in opioid-dependent mothers' fetuses a decreased FHR could be observed in that time period.

### Use of methadone in infancy

Drug dependence is dangerous in infancy. Babies of opioid-dependent mothers have a higher risk of mortality in their first year of life (Burns et al., 2010[16]). Occurrence of sudden infant death syndrome has also been reported in newborns of mothers on MMT. Although there is transient benign increase in QT interval up to 500 msec in healthy newborns, maternal methadone use prolongs QTc in infants in the first two days of their life, which is similar to the effects of methadone in adults (Nekhayeva et al., 2005[108]; Parikh et al., 2011[112]; Philipp et al., 2003[120]; Villain et al., 1992[157]). Hussain and Ewer (2007[58]) presented a case of significant QT interval prolongation in a neonate secondary to use of methadone by its mother. They showed that methadone, even in low maternal doses, could cause conduction disturbances in neonates. Parikh et al. (2011[112]), compared QTc interval in infants born to mothers on MMT with healthy controls. In the first group, QTc interval was significantly prolonged on days 1 and 2 of life. On days 4 and 7, this increase was no longer present. It was concluded that maternal methadone therapy could prolong QTc in newborns and infants and put them at risk of cardiac rhythm disturbances. Wheeler and Tobias (2006[163]) reported a case of methadone use in an infant whose heart rate decreased from 130-140 beats/min to 80-90 beats/min after the first dose of methadone concluding that methadone had the same structure as calcium channel antagonists and led to bradycardia.

As previously shown, elimination of methadone from the body is slower in neonates compared with older children or adults. Methadone is greatly lipid-soluble and highly protein-bound which is responsible for its larger volume of distribution and slower clearance. In fact, neonates have more lipid tissue and higher plasma protein concentrations, which may change drug distribution and metabolism significantly (Chana and Anand, 2001[20]).

### Use of methadone in children

Methadone is currently more available in American houses which predisposes the toddlers to its toxicity (Boyer et al., 2010[15]). Opioids are often used as sedatives in pediatric intensive care unit (PICU). Administration of methadone in PICU was first reported in 1990 to prevent opioid withdrawal (Siddappa et al., 2003[143]); however, it is now increasingly being administered as a treatment of chronic pain syndromes in children (Boyer et al., 2010[15]; Wood et al., 2002[166]). There have also been case reports of methadone use to prevent abstinence syndrome in the PICU and for the treatment of burns in children (Davies et al., 2008[30]). The factors that have made methadone attractive in pediatrics include its comfortable oral use, longer half-life, and ease in calculation of the dose because of its equivalent potency with morphine (Siddappa et al., 2003[143]). 

Although methadone use is beneficial in pediatrics, it has known toxic effects on cardiac conduction that may worsen medical problems appearing in puberty (Boyer et al., 2010[15]; Katchman et al., 2002[72]). Children are particularly susceptible to effects from QT prolongation (Boyer et al., 2010[15]). Yet, in some cases, there is a transient and benign QT prolongation in apparently healthy children (Hussain and Ewer, 2007[58]). Pediatric methadone toxicity in children has been shown to cause peripheral vasodilation (predisposing to orthostatic hypotension), sinus bradycardia, and cerebrovascular vasodilatation (due to decreased sensitivity of the respiratory center to CO_2_ leading to an increased PCO_2_), which can increase intracranial pressure. No arrhythmias such as Tdp are described (Guay, 2009[52]).

### Animal studies

In animals, opioids are used as part of balanced anesthetic methods and for treating pain (Maiante et al., 2009[94]; Ingvast‐Larsson et al., 2010[59]). However, there is little information about the safety and analgesic efficacy of methadone in veterinary medicine (Maiante et al., 2009[94]).

There are several studies about pharmacology of methadone in dogs (Ingvast‐Larsson et al., 2010[59], 2007[60]; Maiante et al., 2009[94]; Pimentel and Mayo, 2008[121]). Elimination of methadone in canines is different from humans. Methadone is rapidly eliminated from the animal body because of a high clearance rate. The oral bioavailability is low in dogs with half-lives of 1.75-6 hours and 2-12 hours following intravenous (IV) and subcutaneous (SC) administration, respectively (Ingvast‐Larsson et al., 2010[59]). Methadone causes cardiovascular depression in dogs. Maiante et al. (2009[94]), compared the effect of methadone and morphine in dogs. Methadone was concluded to induce depressant dose-related cardiovascular changes in conscious dogs. In fact, methadone causes a dose-dependent decrease in heart rate and cardiac index as well as increase in the systemic vascular resistance index. Garofalo and colleagues (2009[49]) compared the cardio-respiratory and neuro-hormonal effects of methadone in conscious and in isoflurane-anaesthetized dogs. Methadone induced dysphoria in all conscious dogs and significantly increased mean arterial pressure, catecholamines, and vasopressin concentrations. During anesthesia, in addition to greater decreases in heart rate and cardiac index, methadone induced apnea and mechanical ventilation was necessary in all dogs. In anaesthetized animals, methadone administration significantly increased vasopressin concentrations and systemic vascular resistance index, while mean arterial pressure did not differ from baseline. In contrast, isoflurane enhanced the intensity of the cardio-respiratory changes induced by methadone. Vasoconstrictive responses associated with methadone did not appear to be induced by vasopressin. Increases in circulating catecholamines, possibly caused by dysphoric reactions to methadone administration, may attenuate the negative chronotropism and the decrease in cardiac index observed when methadone is administered to conscious dogs.

Methadone use increases plasma vasopressin up to 40 times in dogs and goats (Garofalo et al., 2012[49]; Ingvast‐Larsson et al., 2010[59]; Pimentel and Mayo, 2008[121]). Vasopressin released by the pituitary stimulates V1a receptors leading to peripheral vasoconstriction. This has been suggested as the possible responsible cause of systemic vasoconstriction detected after methadone administration (Garofalo et al., 2012[49]).

It was shown that high methadone doses affected cardiac function in guinea pig heart cell preparations. Methadone strengthens the inotropic heart response to sympathetic stimulation in a dose-dependent manner that can be antagonized by increased extracellular calcium concentration (Anguera et al., 2008[8]; Gil et al., 2003[50]; Lamont and Hunt, 2006[86]).

At concentrations of ≥ 10 μM in isolated sheep Purkinje fibers, methadone decreases the maximum rate of depolarization and increases the duration of action potential. High concentrations of methadone can affect several parameters of cardiac function through a mechanism different from opioid receptor stimulation causing QT prolongation (Gil et al., 2003[50]).

### Treatment

The treatment of methadone cardiotoxicity depends on patients' signs and symptoms. The asymptomatic or symptomatic prolongation of QTc interval are differently managed (Deamer et al., 2001[33]). In the case of prolonged QT syndrome and Tdp, predisposing factors should be corrected and use of special medications should be evaluated (Nair et al., 2008[106]). If an increased serum methadone concentration is the reason of cardiotoxicity, it is advised to lower the methadone dose (Krook et al., 2004[83]). Methadone-induced Tdp associated with hypokalemia should be treated with IV potassium. Magnesium administration is suggested for those with QTc prolongation even if the serum concentration of Mg is normal (Deamer et al., 2001[33]; Zipes et al., 2006[168]). Since R-isomer less inhibits the hERG channel, substitution of *(R,S)*-methadone by *(R)-*methadone reduces the QTc interval value (Ansermot et al., 2010[9]). If the patient has a QTc ≥ 500 msec, it is reasonable to change methadone with another drug and refer the patient to a cardiologist (Somberg and Molnar, 2002[145]). The proper alternatives include buprenorphine, naltrexone, or slow-release oral morphine (Deamer et al., 2001[33]; Kastelic et al., 2008[71]).

Buprenorphine is a partial μ-agonist and a κ-antagonist effective at lowering the use of opioids among opiate-addicted people. No report of QT prolongation has been given with buprenorphine. Thus, it is a good alternative for methadone in opiate dependency and chronic pain (Esses et al., 2008[39]). During the first years of methadone and buprenorphine administration, death rate of these two drugs was compared concluding that buprenorphine was associated with a much lower mortality rate than methadone (Perrin‐Terrin et al., 2011[119]). A 56-year-old man with syncope and Tdp secondary to methadone has been presented in the literature in whom, after transition to buprenorphine, QT interval normalized and ventricular arrhythmias resolved (Esses et al., 2008[39]). De Jong and De Ruiter (2013[32]) introduced a 52-year-old man who was admitted to the hospital due to Tdp. He had used methadone two days earlier. Buprenorphine was substituted and QTc normalized after two weeks. In a cross-sectional study by Fanoe and colleagues (2007[41]), methadone was associated with long QT interval, but there was not any association between buprenorphine and QTc interval. Stallvik et al. (2013[148]), found that buprenorphine was a suitable alternative for methadone regarding the risk of QTc prolongation.

In cases of recurrent methadone-associated Tdp whose medication cannot be changed, permanent pacing with the use of defibrillator Implantable Cardioverter Defibrillator (ICD) can be done (Miller et al., 2011[104]). Patel and coworkers (2008[115]) reported that from their eight patients undergoing this treatment, three who continued taking methadone after ICD placement received shocks for Tdp within the 2-year follow-up. The procedure was therefore heralded as potentially lifesaving for people with a history of Tdp who continued to take methadone. However, one of the eight patients died because of unknown causes, and two suffered serious peri-operative complications (pericardial tamponade and device infection).

Another choice of treatment in these patients with limitations in switching the drug or those at risk of device infection is left cardiac sympathetic denervation (LCSD). This method is a preganglionic denervation with antifibrillatory effects and emerging as an important adjunctive therapy in the management of ventricular arrhythmias. Miller et al. (2011[104]), introduced the first case of drug-induced QT prolongation successfully managed with LCSD. 

The efficacy of oral activated charcoal for adsorption of drugs and poisons has been widely described in the literature. Activated charcoal is helpful if administrated within 1-2 hours after ingestion. Since routine use of AC is discouraged, it is important to consider the risks and benefits of AC on a drug-by-drug basis (Chyka et al., 2005[24]; Khosrojerdi et al., 2013[75]).

### Recommendations

MMT personnel, patients, and their families need to be better informed about the manifestations of methadone poisoning and their suitable management to reduce the morbidity and mortality rates associated with methadone (Caplehorn and Drummer, 2002[19]). It is recommended that methadone patients and their families be evaluated regarding any history of syncope, sudden death, structural heart disease, or any other cardiac-related signs and symptoms. In addition, physicians should seek for the use of agents inhibiting CYP3A4 or CYP2B6 enzymes especially cimetidine, ciprofloxacin, erythromycin, clarithromycin, fluvoxamine, and pink grapefruit or Seville orange juice (Anderson and Kearney, 2000[7]; Boyer et al., 2010[15]). Clinicians prescribing methadone should also notice the wide inter-individual variability in the rate of methadone metabolism and the small difference between its therapeutic and toxic doses (Backmund et al., 2001[13]; Mehrpour et al., 2013[102]).

Before administration of methadone, conducting cardiovascular screening is reasonable and cheap. Screening includes echocardiography (to exclude heart disease) and check of possible electrolyte imbalances (Justo et al., 2006[67]). ECG assessment might be considered in new patients with history of known heart disease or recent symptoms like unexplained seizures, exertional chest pain or discomfort, exertional dyspnea, unexplained syncope or heart palpitations (Maremmani et al., 2004[96]). QT interval calculation should be conducted before prescription of methadone. The ECG should be checked at regular intervals. This is especially important within days of starting methadone therapy (Krook et al., 2004[83]); the ECG should be obtained before initiation of methadone and 4-7 days after its initiation. Additional ECGs are recommended 4-7 days after increase of dosage, when patients have unexplained syncope or seizures, and/or when there are changes in condition or therapy, which increase the risk of arrhythmia (Deamer et al., 2001[33]). ECG screening may not be possible in all patients on MMT. Although the screening procedure itself is not expensive, interpretation of ECGs by cardiologists may be expensive (Fareed et al., 2010[42]). Automated measurement of the QT interval may be used but is not accurate or trustworthy enough to be applied (Calver et al., 2012[18]; Isbister et al., 2009[61]; Malik and Camm, 2001[95]).

In order to better show the effect of methadone on the QT interval over a 24-hour period, high-resolution digital holter recorders are the most accurate tools to measure the QT with modern algorithms. Calver et al. (2012[18]), found that this method could make a risk assessment; however, this still required manual interpretation of the QT using on-screen magnification and calipers (Hnatkova et al., 2006[56]).

## Conclusion

Methadone has become popular in the treatment of opioid addiction and pain because of its special pharmacokinetic and pharmacodynamic characteristics. But, patients are at risk of methadone cardiotoxicity. Effectiveness of methadone in the treatment of pain and addiction should be weighed against these adverse effects and physicians should consider the ways to minimize these undesirable effects.

## Conflict of interest

Authors declare no conflict of interest.

## Acknowledgement

This article is supported by Atherosclerosis and Coronary Artery Research Center in Birjand University of Medical Sciences. It is M.D thesis of the first author.

## Figures and Tables

**Table 1:**
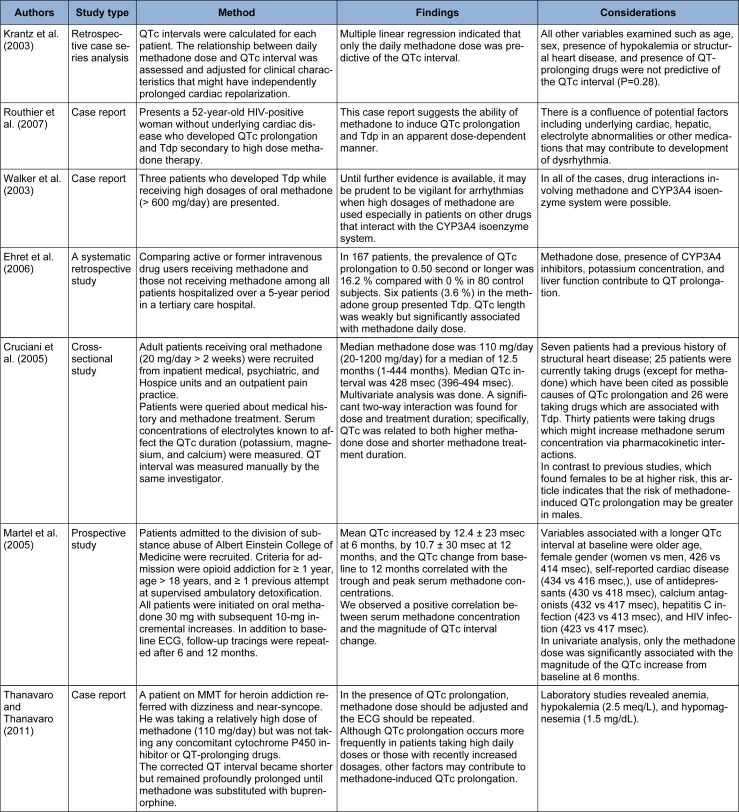
Positive dose-response relationship between methadone and cardiotoxicity

**Table 2:**
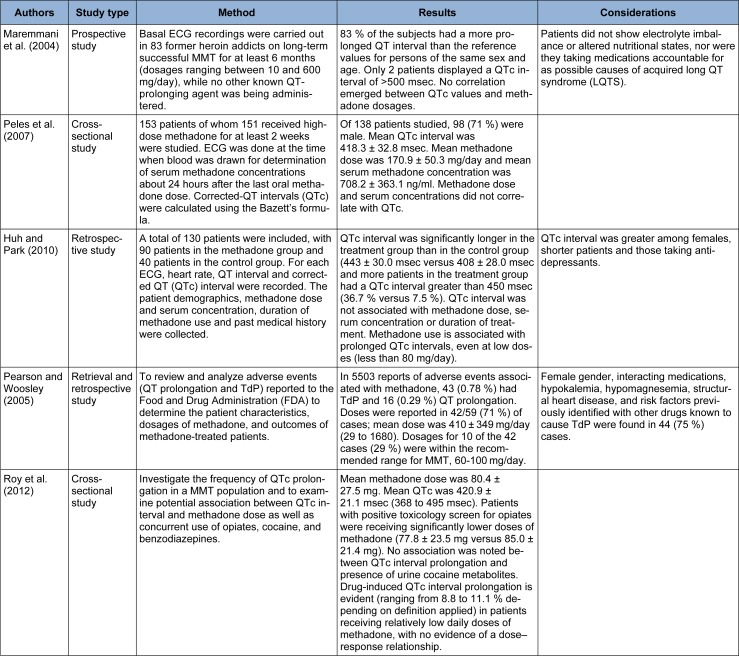
Negative dose-response relationship between methadone and cardiotoxicity
